# New *Mycobacteroides abscessus* subsp. *massiliense* strains with recombinant *hsp65* gene laterally transferred from *Mycobacteroides abscessus* subsp. *abscessus*: Potential for misidentification of *M. abscessus* strains with the *hsp65*-based method

**DOI:** 10.1371/journal.pone.0220312

**Published:** 2019-09-13

**Authors:** Byoung-Jun Kim, Ga-Na Kim, Bo-Ram Kim, Tae-Sun Shim, Yoon-Hoh Kook, Bum-Joon Kim

**Affiliations:** 1 Department of Microbiology and Immunology, Biomedical Sciences, Liver Research Institute and Cancer Research Institute, College of Medicine, Seoul National University, Seoul, Korea; 2 Division of Pulmonary and Critical Care Medicine, Department of Internal Medicine, University of Ulsan College of Medicine, Asan Medical Center, Seoul, Korea; National Institute of Infectious Diseases, JAPAN

## Abstract

It has been reported that lateral gene transfer (LGT) events among *Mycobacteroides abscessus* strains are prevalent. The *hsp65* gene, a chronometer gene for bacterial phylogenetic analysis, is resistant to LGT events, particularly among mycobacterial strains, rendering the *hsp65-*targeting method the most widely used method for mycobacterial detection. To determine the prevalence of *M*. *abscessus* strains that are subject to *hsp65* LGT, we applied *rpoB* typing to 100 clinically isolated Korean strains of *M*. *abscessus* that had been identified by *hsp65* sequence analysis. The analysis indicated the presence of 2 rough strains, showing a discrepancy between the 2 typing methods. MLST analysis based on the partial sequencing of seven housekeeping genes, *erm*(41) PCR and further *hsp65* PCR-restriction enzyme and polymorphism analysis (PRA) were conducted to identify the two strains. The MLST results showed that the two strains belong to *M*. *abscessus* subsp. *massiliense* and not to *M*. *abscessus* subsp. *abscessus*, as indicated by the *rpoB*-based analysis, suggesting that their *hsp65* genes are subject to LGT from *M*. *abscessus* subsp. *abscessus*. Further analysis of these strains using the *hsp65* PRA method indicated that these strains possess a PRA pattern identical to that of *M*. *abscessus* subsp. *abscessus* and distinct from that of *M*. *abscessus* subsp. *massiliense*. In conclusion, we identified two *M*. *abscessus* subsp. *massiliense* rough strains from Korean patients with *hsp65* genes that might be laterally transferred from *M*. *abscessus* subsp. *abscessus*. To the best of our knowledge, this is the first demonstration of possible LGT events associated with the *hsp65* gene in mycobacteria. Our results also suggest that there is the potential for misidentification when the *hsp65*-based protocol is used for mycobacterial identification.

## Introduction

Rapidly growing mycobacteria (RGM) are ubiquitous organisms that have gained increasing attention as important human pathogens [1, 2]. Among RGMs, infections due to the *Mycobacteroides abscessus* strains have shown increased worldwide clinical importance, and their incidence in cystic fibrosis patients has increased [3]. In South Korea, the incidence of lung diseases caused by *M*. *abscessus* has also been increasing, and this organism accounts for 70–80% of the lung disease caused by RGM [4–7]. *M*. *abscessus* can cause lung disease in immunocompetent individuals and shares a number of characteristics with *M*. *tuberculosis*, including the ability to induce granulomatous lesions or caseous necrosis [8]. Infections involving *M*. *abscessus* are notorious for being difficult to treat due both to the natural broad-spectrum antibiotic resistance of this species and to its acquired resistance, with disparate antibiotic susceptibility patterns being observed among clinical strains [9].

The taxonomy of *M*. *abscessus* strains remains problematic. Currently, these strains are divided into two subspecies, *M*. *abscessus* subsp. *abscessus* (the former species *Mycobacteroides abscessus*) and *M*. *abscessus* subsp. *bolletii*. *M*. *abscessus* subsp. *bolletii* was proposed to combine the two former species, *M*. *massiliense* and *M*. *bolletii* [10, 11]. *M*. *massiliense* can be further subdivided into two genotypes (Type I and Type II) based on *hsp65* sequence analysis [12–14]. Also, recent phylogenomics and comparative genome analyses on 150 genomes of *Mycobacterium* species revealed that the genus of *Mycobacterium* was divided four novel genera. In the case of *M*. *abscessus-chelonae* complex, their genus was emended into *Mycobacteroides* [15].

Lateral gene transfer (LGT) has been proposed as the major driving force for the acquisition of prokaryotic genetic diversity, an attribution that leads to better survival of prokaryotic organisms under harsh environmental conditions [16, 17]. The recent increase in available information on mycobacterial genomes supports the idea that LGT plays an important role in the evolutionary transition of mycobacteria from saprophytic organisms into opportunistic or specialized, highly persisting pathogens [18, 19]. In particular, interspecies or intraspecies LGT events among members of the *M*. *abscessus* strains have been reported to be very prevalent [20]. And it has reported that *M*. *abscessus* evolution is sporadically punctuated by dramatic genome wide remodelling events [21]. We recently identified six *M*. *abscessus* subsp. *massiliense* strains isolated from Korean patients in which the *rpoB* gene was laterally transferred from *M*. *abscessus* subsp. *abscessus* [22], suggesting the potential for misidentification when the *rpoB* tying method is used to the differentiate among *M*. *abscessus* strains. In addition, other reports have presented evidence for LGT events involving the *rpoB* gene, including the observation that the *rpoBC* operon of the Type I genotype of *M*. *yongoenense* has been laterally transferred from a distantly related strain, *Mycobacterium parascrofulaceum* [23].

The *hsp65* (groEL2) gene, another chronometer molecule, has been widely used as a targeting molecule for mycobacterial identification and detection [24, 25] together with the *rpoB* gene [26, 27]. In this study, we sought to address issues associated with the possibility that LGT events involving the *hsp65* gene occur among members of the *M*. *abscessus* strains. To this end, we applied *rpoB* typing (711 bp) to 100 clinically isolated Korean strains of *M*. *abscessus* that had already been identified by *hsp65* sequence analysis (603 bp). In the strains in which the two methods yielded discordant results, additional phylogenetic analysis was conducted to confirm the authenticity of potential LGT events in their *hsp65* genes.

## Materials and methods

### Mycobacterial strains and culture conditions

Of the total 206 clinical isolated strains, 106 strains were used in the previous paper. These strains were identified as *M*. *massiliense* using the *hsp65*-based method. Among these 106 strains, 6 strains showed different *rpoB* sequence from *M*. *massiliense*, however, these strains showed similar sequence homologies with *M*. *massiliense*. So, the possibility of lateral gene transfer in the *rpoB* gene of these strains were described in the previous study [22].

We used other 100 strains of the total 206 strains in this study which were identified as *M*. *abscessus* using the *hsp65*-based method [12] (Table 1). These *M*. *abscessus* strains included both rough and smooth morphotypes (28 and 72 strains, respectively). Among them, two strains, 55262 and 55184 showed different *rpoB* gene sequence from *M*. *abscessus*. Although both studies used samples collected at the same time period and applied similar experimental methods, the two studies were considered to be different, because different groups of samples (which were divided by *hsp65*-based method) were used and putative lateral gene transfer events were considered to be applied to different genes, respectively.

**Table 1:** Separation of 100 *M*. *abscessus subsp*. *abscessus* clinical strains into genotype level by sequence analyses based on the partial *hsp65* (603 bp) and *rpoB* (711 bp) gene sequences.

Sequence identification	*M*. *abscessus* subsp. *abscessus*	*M*. *abscessus* subsp. *massiliense*
*hsp65*-based results (No., %)	100 (100.0)	0 (0.0)
*rpoB*-based results (No., %)	98 (98.0)	2 (2.0)

All clinical strains were collected from the Asan Medical Center (Seoul, Republic of Korea) from January 2004 to June 2011. This work was approved by the institutional review board of the Asan Medical Center (2012–0170) with documentation for waivers of informed consent. Each bacterial isolate was maintained on Middlebrook 7H10 agar plates supplemented with OADC or in Middlebrook 7H9 broth medium supplemented with ADC at 37°C. The bacteria were stored as frozen stocks at -70°C by flash-freezing of intermediate passage samples in 20% glycerol. *M*. *abscessus* subsp. *abscessus* ATCC 19977^T^ (= CIP 104536^T^), *M*. *abscessus* subsp. *bolletii* CIP 108541^T^ and *M*. *abscessus* subsp. *massiliense* CIP 108297 (= CCUG 48898) were also used as type strains.

### DNA extraction, PCR and sequencing

Bacterial DNA was extracted from individual clinical isolates using the bead beater-phenol extraction method [24] and used as templates for PCR amplification. Partial *hsp65* (603 bp) and *rpoB* (711 bp) gene-targeted PCR was conducted in a total of 100 *M*. *abscessus* strains as described previously [12, 24]. MLST analyses targeting seven housekeeping genes were applied to investigate the genetic diversity of the two putative recombinant strains. The seven target genes were *argH* (argininosuccinate lyase), *cya* (adenylate cyclase), *glpK* (glycerol kinase), *gnd* (6-phosphogluconate dehydrogenase), *murC* (UDP N-acetylmuramate-L-alanine ligase), *pta* (phosphate acetyltransferase) and *purH* (phosphoribosylaminoimidazole carboxylase ATPase subunit) [28, 29]. Also, *erm*(41)-targeted PCR was applied to the reference strain and to two Rec-mas-H strains [30, 31]. Additionally, to confirm the putative recombination site of the Rec-mas-H strain within the *hsp65* gene sequence, the entire *hsp65* gene sequence of Asan 55262 was sequenced and compared with the *hsp65* gene sequences of the *M*. *abscessus* subsp. *abscessus* CIP 104536^T^, *M*. *abscessus* subsp. *massiliense* CIP 108297^T^ and *M*. *abscessus* subsp. *massiliense* Asan 50594 strains. The complete *hsp65* gene sequence of each selected isolate was amplified using 5 primer sets. Detailed information on the primers is provided in S1 Table. All the PCR reactions were conducted as described previously [22].

### PCR-based restriction analysis

The partial *hsp65* gene sequence was amplified using the primer set Tb11 (5’- ACCAACGATGGTGTGTCCAT-3’) and Tb12 (5’- CTTGTCGAACCGCATACCCT-3’) as described by Telenti *et al* [25]. Restriction analyses were performed as described by Telenti *et al* [25]. Briefly, 10 μl of final amplicons were digested with *BstE*II (NEB, Ipswich, MA, UK; 37°C) and *Hae*III (Takara Bio, Shiga, Japan; 60°C) for 2 hours. The two restriction fragments were separated by electrophoresis on 1% agarose gels with a 100-bp DNA ladder as the molecular size standard.

### Sequence analyses

The obtained sequences of *hsp65* (603 bp), *rpoB* (711 bp) and of 7 MLST target genes, such as *argH* (503 bp), *cya* (541 bp), *glpK* (563 bp), *gnd* (494 bp), *murC* (545 bp), *pta* (486 bp) and *purH* (549 bp) from the two putative recombinant strains were aligned with those of *M*. *abscessus* complex type strains using the ClustalW algorithm in the MEGA 4.0 program [32]. Phylogenetic trees based on each target gene or concatenated sequence were constructed by the neighbor-joining [33] and maximum parsimony [34] methods with 1,000 replicates [35].

### Nucleotide sequence accession numbers

The *hsp65*, *rpoB* and 7 MLST gene sequences determined in this study were deposited in GenBank under the accession numbers MH430895—MH430913 and are listed in S2 Table.

## Results

### Identification of two *M*. *abscessus* strains for which discordant results were obtained in *hsp65* and *rpoB* sequence analyses

**Fig 1:**
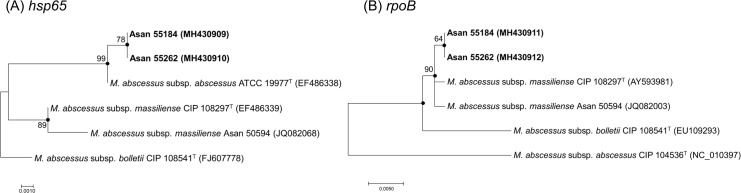
Phylogenetic trees based on the partial *hsp65* and *rpoB* gene sequences of two Rec-mas-H strains. Phylogenetic trees of 2 Rec-mas-H strains based on (A) the partial sequence of the *hsp65* gene (603 bp) and (B) the partial sequence of the *rpoB* gene (711 bp). The trees were constructed using the neighbor-joining method in the MEGA 4.0 program. The bootstrap values were calculated from 1,000 replications; values <50% are not shown. The black-centered circles indicate that the corresponding clusters were supported with maximum parsimony-based trees. The bar indicates the number of base substitutions per site. The black-centered triangles indicate that the corresponding sequences were sequenced and obtained in this study.

Among 100 *M*. *abscessus* strains that were previously identified as *M*. *abscessus* subsp. *abscessus* by 603-bp *hsp65* sequencing analysis for subspecies differentiation of the *M*. *abscessus* strains, two strains (2.0%, 2/100) were identified as *M*. *abscessus* subsp. *massiliense* by 711-bp *rpoB* sequence-based phylogenetic analysis (Table 1). The two Rec-mas-H strains (Asan 55262 and 55184) that yielded results discordant with those obtained by *rpoB*-based analysis had identical *hsp65* sequences, with 1 bp mismatch (99.8% sequence homology) to the *hsp65* sequence of *M*. *abscessus* subsp. *abscessus* type strain, CIP 104536^T^ (Fig 1, Table 1). The results showed that no putative recombination site was present in the compared *hsp65* gene sequences. Only three base pairs (located at 779, 918, and 1296 nt of the 1,626-bp *hsp65* sequence) (S1 Fig) differed between the Asan 55262 and the *M*. *abscessus* subsp. *abscessus* CIP 104536^T^ strains.

### Phylogenetic analysis of two Rec-mas-H strains based on seven different MLST genes

**Fig 2:**
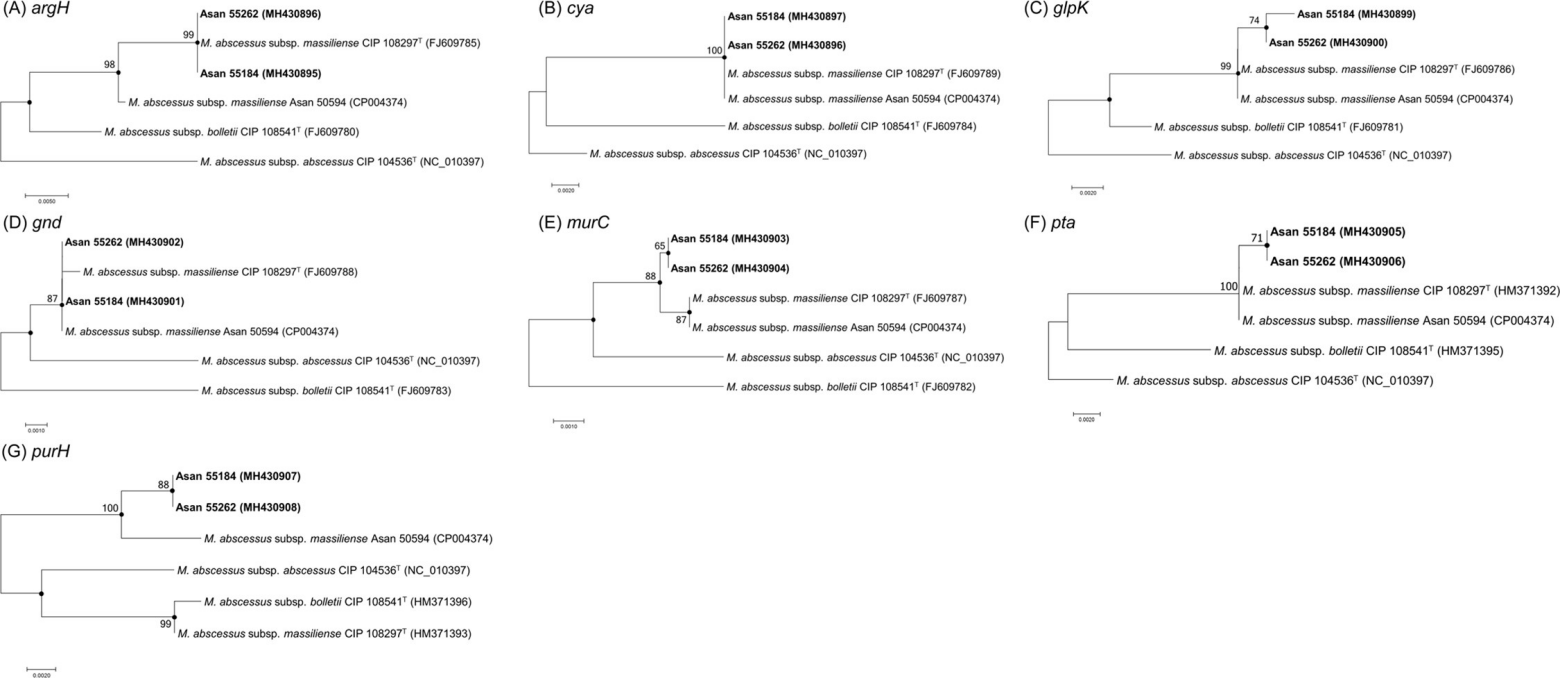
Neighbor-joining phylogenetic trees based on the 7 MLST genes of two Rec-mas-H strains. Phylogenetic trees of two Rec-mas-H strains constructed based on the partial sequencing of six MLST genes. (A) *argH*, (B) *cya*, (C) *glpK*, (D) *gnd*, (E) *murC*, (F) *pta* and (G) *purH* gene sequence based trees were constructed using the neighbor-joining method in the MEGA 4.0 program. The bootstrap values were calculated from 1,000 replications; values <50% are not shown. The black-centered circles indicate that the corresponding clusters were supported by maximum parsimony-based trees. The bar indicates the number of base substitutions per site.

For precise species delineation of the two strains, MLST analyses based on the partial sequencing of seven housekeeping genes, *argH*, *cya*, *glpK*, *gnd*, *murC*, *pta* and *purH*, that have been previously used for the elucidation of recombination events in *M*. *abscessus* strains [29] were also performed in this study. Each single-gene-based tree was built from the sequences of each of the seven genes in the MLST scheme for the separation of the two strains at the subspecies level of the *M*. *abscessus* strains (Fig 2). With the exception of the *glpK* gene, which has a 1-bp difference in the sequence, the sequences of all the MLST genes were identical in the two Rec-mas-H strains. Most of the constructed phylogenetic trees (those for the *argH*, *cya*, *glpK*, *gnd*, *murC*, and *pta* genes) showed a topology similar to that of the *rpoB* gene sequence-based tree. The two Rec-mas-H strains were clustered together with the *M*. *abscessus* subsp. *massiliense* CIP 108297^T^ strain or the *M*. *abscessus* subsp. *massiliense* Asan 50594 strain, which has been described as the Type II genotype of the *M*. *abscessus* subsp. *massiliense* strain [12, 14]. The *argH* and *cya* gene sequences of the two Rec-mas-H strains showed 100% sequence similarity with those of *M*. *abscessus* subsp. *massiliense* CIP 108297^T^ (Table 2, Fig 2A and 2B). However, in the *purH* gene-based tree, the two Rec-mas-H strains were closely clustered with the *M*. *abscessus* subsp. *massiliense* Asan 50594 strain but not with the *M*. *abscessus* subsp. *massiliense* CIP 108297^T^ strain. In addition, the *purH* gene sequence similarity between the two Rec-mas-H strains and *M*. *abscessus* subsp. *massiliense* CIP 108297^T^ showed the lowest value of 97.6% among the seven MLST genes.

**Table 2:** Comparison of two Rec-mas-H strains with reference strains of *M*. *abscessus* group in sequence similarities of 7 MLST, *hsp65* (603 bp) and *rpoB* (711 bp) gene sequences.

Genes (compared size, bp)	Sequence similarities between Rec-mas-H strains and type or reference strains (%)		
	*M*. *abscessus* subsp. *abscessus* CIP 104536^T^	*M*. *abscessus* subsp. *massiliense* CIP 108297^T^	*M*. *abscessus* subsp. *massiliense* Asan 50594
*argH* (503)	95.4	100.0	99.0
*cya* (541)	98.2	100.0	100.0
*glpK* (563)	97.7, 97.9	99.6, 99.8	99.6, 99.8
*gnd* (494)	97.8	99.8	100.0
*murC* (545)	98.0	99.6	99.6
*pta* (486)	97.9	99.8	99.8
*purH* (549)	97.6	97.6	99.1
*hsp65* (603)	99.8	98.7	98.3
*rpoB* (711)	96.5	99.7	99.7
Concatenated 7 MLST genes (3,681)	97.6	99.5	99.6
Concatenated 7 MLST genes + *hsp65* (4,284)	97.9	99.4	99.4
Concatenated 7 MLST genes + *rpoB* + *hsp65* (4,995)	97.7	99.4	99.5

With the exception of the *purH* tree, all of the MLST gene-based trees (*argH*, *cya*, *glpK*, *gnd*, *murC* and *pta* genes) indicated that the two strains were closely related to the *M*. *abscessus* subsp. *massiliense* group, and these topologies were strongly supported by analysis based on the maximum parsimony algorithm (Fig 2). The calculated sequence similarities of all 9 genes (7 MLST genes, the *hsp65* gene and the *rpoB* gene) and their concatenated sequences between the reference strains of the *M*. *abscessus* strains and two Rec-mas-H strains are shown in Table 2 and S3 Table.

### Phylogenetic analysis of two Rec-mas-H strains using trees based on concatenated sequences

**Fig 3:**
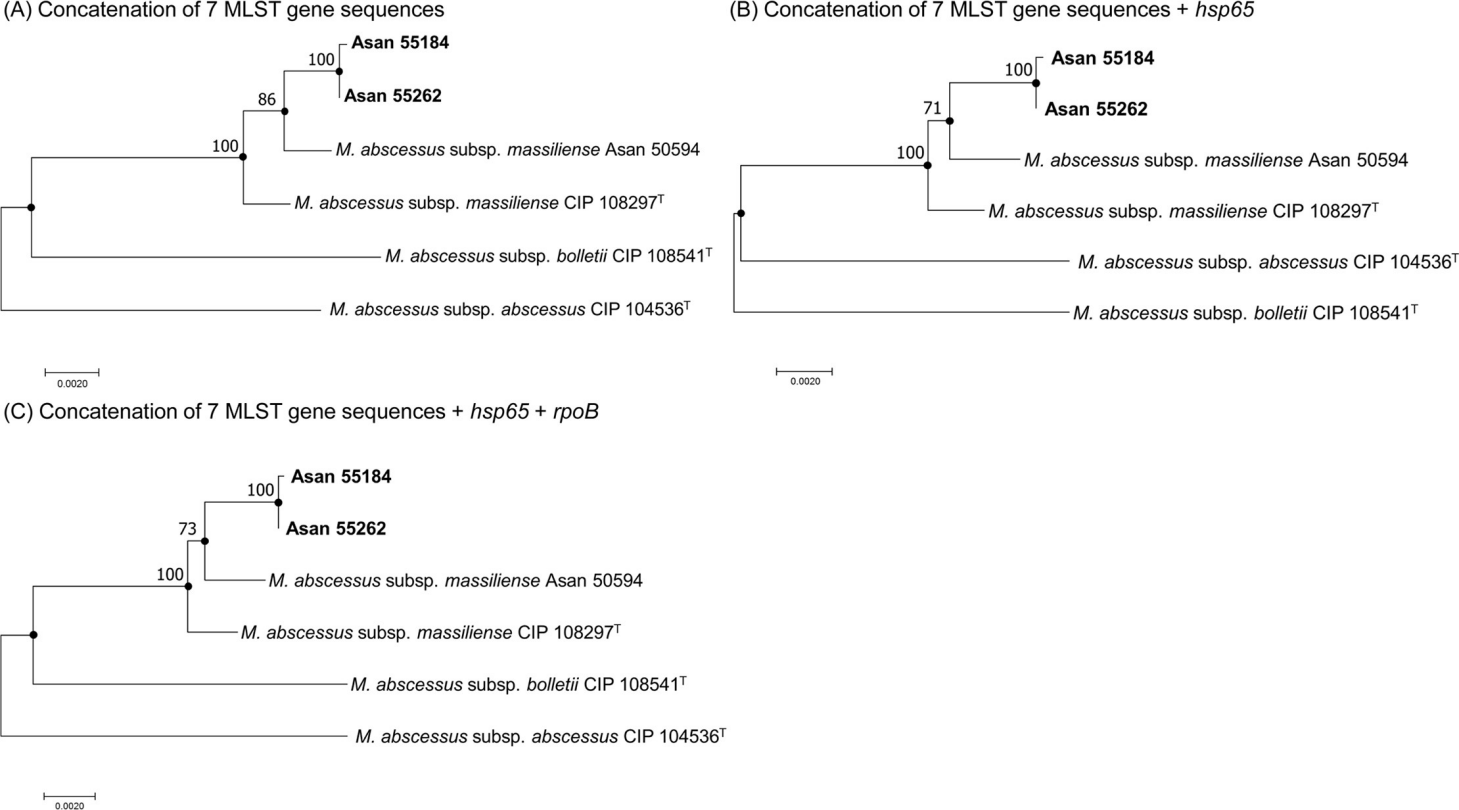
Neighbor-joining phylogenetic trees based on the concatenated sequences of two Rec-mas-H strains. Phylogenetic trees based on (A) concatenation of 7 MLST gene sequences, (B) concatenation of 7 MLST gene sequences and the *hsp65* gene sequence, and (C) concatenation of 7 MLST gene sequences and the *hsp65* and *rpoB* gene sequences. The trees for all studied strains were generated using the neighbor-joining method. The bootstrap support values (%) from 1,000 replications are indicated for each node; values <50% are not shown. The black-centered circles indicate that the corresponding clusters were supported by maximum parsimony-based trees. The bar indicates the number of base substitutions per site.

The phylogenetic tree based on the concatenated sequences of the seven MLST genes showed that the two Rec-mas-H strains belong to the *M*. *abscessus* subsp. *massiliense* group and not to *M*. *abscessus* subsp. *abscessus* or *M*. *abscessus* subsp. *bolletii* (Fig 3A) as shown in the *rpoB*-based analysis (Fig 1B). This suggests that the Rec-mas-H strains may be members of *M*. *abscessus* subsp. *massiliense* rather than *M*. *abscessus* subsp. *abscessus* and that their *hsp65* gene may have undergone lateral gene transfer from *M*. *abscessus* subsp. *abscessus*. In the tree based on the concatenated sequences of the seven MLST genes, the two Rec-mas-H strains were closer to the *M*. *abscessus* subsp. *massiliense* Asan 50594 strain than to the *M*. *abscessus* subsp. *massiliense* type strain; this result may be due to the differences in the *purH* gene sequence (Fig 2G, Table 2 and S3 Table).

Addition of the *hsp65* and/or *rpoB* gene sequences to the 7 MLST concatenated gene sequences did not affect the overall topology of the tree obtained using the 7 MLST concatenated sequences (Fig 3B and 3C). However, the addition of the *hsp65* gene sequence slightly affected the sequence similarity between the two Rec-mas-H strains and the *M*. *abscessus* subsp. *abscessus* and *M*. *abscessus* subsp. *massiliense* group strains. The sequence similarity with *M*. *abscessus* subsp. *abscessus* was slightly increased (from 97.6–97.7% to 97.9%), and the similarity with *M*. *abscessus* subsp. *massiliense* group strains was slightly decreased (from 99.5–99.6% to 99.4%) after addition of the *hsp65* gene sequence (Table 2).

### Separation of 2 Rec-mas-H strains by *erm*(41) PCR at the subspecies level

**Fig 4:**
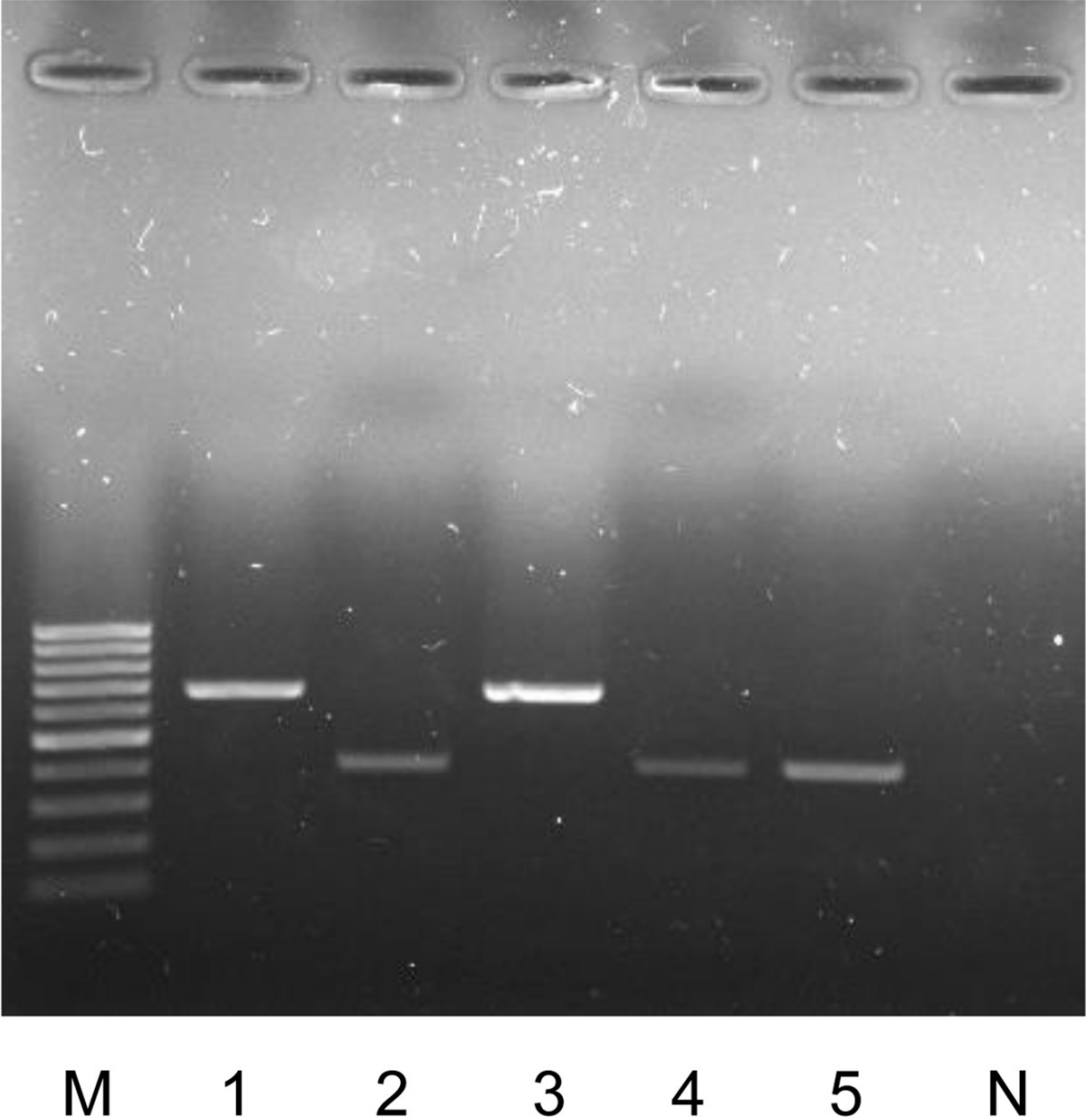
Identification of two Rec-mas-H strains at the subspecies level by PCR targeting the *erm*(41) gene. M, 100-bp DNA ladder; Lane 1, *M*. *abscessus* subsp. *abscessus* CIP 104536^T^; Lane 2, *M*. *abscessus* subsp. *massiliense* CIP 108297^T^; Lane 3, *M*. *abscessus* subsp. *bolletii* CIP 108541^T^; Lane 4, Asan 55814; Lane 5, Asan 55262; N, negative control.

The *M*. *abscessus* subsp. *massiliense erm*(41) gene is reported to have a large C-terminal deletion. Therefore, *erm*(41) PCR can be used as a simple method to differentiate *M*. *abscessus* subsp. *massiliense* from *M*. *abscessus* subsp. *abscessus* and *M*. *abscessus* subsp. *bolletii* species [30, 31]. To further confirm the authenticity of the two Rec-mas-H strains, we applied *erm*(41) PCR; unlike the *M*. *abscessus* subsp. *abscessus* and *M*. *abscessus* subsp. *bolletii* type strains, which produced a full-sized product (approximately 700 bp), the two Rec-mas-H strains produced a shorter product (approximately 350 bp) that was identical to the product of *M*. *abscessus* subsp. *massiliense* type strain (Fig 4).

### Differentiation of 2 Rec-mas-H strains from *M*. *abscessus* subsp. *massiliense* based on restriction patterns of the partial *hsp65* gene sequence

**Fig 5:**
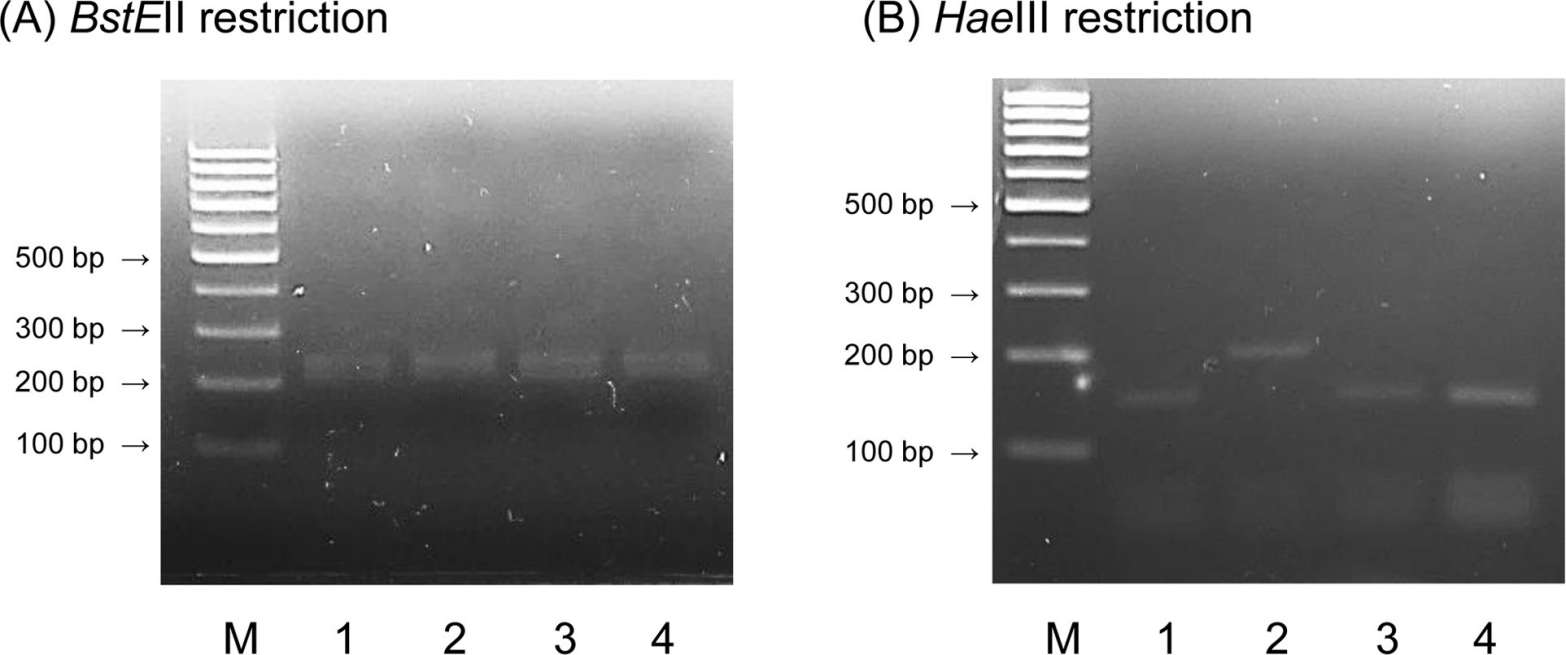
Differentiation of two Rec-mas-H strains by PCR-restriction enzyme and polymorphism analysis (PRA) of *hsp65*. Amplified *hsp65* gene amplicons were digested with (A) *BstE*II and (B) *Hae*III restriction enzymes. M, 100-bp DNA ladder; Lane 1, *M*. *abscessus* subsp. *abscessus* CIP 104536^T^; Lane 2, *M*. *abscessus* subsp. *massiliense* CIP 108297^T^; Lane 3, Asan 55814; Lane 4, Asan 55262.

PCR-based restriction enzyme and polymorphism analysis (PRA) was performed for further differentiation of the 2 Rec-mas-H strains from *M*. *abscessus* subsp. *massiliense*. Using the two restriction enzymes, *BstE*II and *Hae*III, the partial *hsp65* gene sequence was digested [25], and the obtained fragments were compared to those obtained fragments from the partial *hsp65* gene sequences of *M*. *abscessus* subsp. *abscessus* and *M*. *abscessus* subsp. *massiliense* by gel electrophoresis. All the *BstE*II restriction digests showed a similar pattern of fragments 220/245 bp in size. However, *Hae*III restriction digests of the 2 Rec-mas-H strains showed patterns identical to those of *M*. *abscessus* subsp. *abscessus* (160/60 bp) but distinct from those of *M*. *abscessus* subsp. *massiliense* (200/60 bp) (Fig 5).

## Discussion

The occurrence of LGT events among species or subspecies in genes encoding chronometer molecules that are used for the diagnosis or identification of pathogenic bacteria may compromise the results obtained when attempting to identify the disease-causing organisms present in infected patients, potentially leading to treatment failure. This problem is especially true in the case of NTM infections, which require a long culture period and often show species- or subspecies-dependent disparities in treatment regimens. Therefore, investigation of LGT events that may affect the diagnosis or identification of target molecules used in the differentiation of mycobacteria is necessary.

The general molecular target for differentiation among species or subspecies in bacterial taxonomy is the 16s rRNA gene [36–38]. However, 16s rRNA sequence-based diagnostic and taxonomic methods have some limitations for species differentiation within the genus *Mycobacterium*, mainly due to the lack of 16S rRNA sequence diversity [39–41]. In particular, differentiation of RGM species, including the *M*. *abscessus* strains, is almost impossible. Therefore, instead of the 16s rRNA gene, alternative chronometer molecules such as the *rpoB* and *hsp65* genes have been widely used as targets for mycobacterial identification. However, LGT events associated with the *rpoB* gene have been reported to occur among mycobacteria species or subspecies. For example, the *M*. *yongonense* Type I strain carries an *rpoBC* operon that was laterally transferred from a distantly related species, *M*. *parascrofulaceum* [23, 42]. In addition, we recently isolated six *M*. *abscessus* subsp. *massiliense* strains from Korean patients with specific hybrid *rpoB* genes that were laterally transferred from *M*. *abscessus* subsp. *abscessus* [22]. This suggests that there is a risk of mis-identification when *rpoB*-based methods are used in mycobacterial diagnosis.

To date, no LGT events associated with another chronometer gene that is used as a mycobacterial target, *hsp65*, have been reported either in mycobacterial strains or in species within other genera, suggesting that the *hsp65* gene is more resistant to LGT than the *rpoB* gene. However, in this study, we identified for the first time two *M*. *abscessus* subsp. *massiliense* rough strains with *hsp65* genes that might have been laterally transferred from *M*. *abscessus* subsp. *abscessus*. These results suggest that the use of *hsp65*-based diagnosis in mycobacteria also creates a risk of misidentification, at least when attempting to differentiate subspecies within the *M*. *abscessus* strains. Indeed, we verified that two *hsp65* recombinant *M*. *abscessus* subsp. *massiliense* strains were mis-identified as *M*. *abscessus* subsp. *abscessus* by the *hsp65*-PRA method targeting the 441-bp Telenti fragment, the most widely used method for NTM differentiation [25]. To the best of our knowledge, this is the first reported case of NTM misidentification by the *hsp65*-PRA method, and it strongly supports the above notion.

In contrast to the phylogenetic similarity of six recombinant *M*. *abscessus* subsp. *massiliense* strains carrying a hybrid *rpoB* gene to the smooth *M*. *abscessus* subsp. *massiliense* Type I genotype reported in our previous study [22], our phylogenetic analysis based on MLST sequences indicated that the two *M*. *abscessus* subsp. *massiliense* strains with recombinant *hsp65* genes from *M*. *abscessus* subsp. *abscessus* are more closely related to *M*. *abscessus* subsp. *massiliense* Type II than to Type I. The results suggest that these two strains may have descended from the *M*. *abscessus* subsp. *massiliense* Type II genotype. Given that all Type II strains have a rough morphotype due to deletion of the glycopeptidolipid (GPL) gene, the rough colony morphotypes of the 2 *hsp65* recombinant strains also support the above notion. However, the two putative recombinant strains showed unique sequences in *hsp65*, *rpoB*, *glpK*, *murC*, *pta* and *purH* genes which were differentiated from *M*. *abscessus* subsp. *massiliense* Type II strain (Figs 1 and 2, S3 Table).

Combined analysis of the results obtained in the present study and in our previous study [22] indicated that LGT events occurred in a total of 8 (3.9%) strains (2 strains with *hsp65* recombination and 6 strains with *rpoB* recombination) in a sample of 206 *M*. *abscessus* strains from Korean patients (100 strains in the present study and 106 strains in the recent study). Of note, further MLST analysis showed that all these strains belonged to the *M*. *abscessus* subsp. *massiliense* subspecies and were not *M*. *abscessus* subsp. *abscessus*, suggesting that *M*. *abscessus* subsp. *massiliense* may be more vulnerable to LGT events than *M*. *abscessus* subsp. *abscessus*. This provides a possible explanation of the fact that the genetic and taxonomic diversity among *M*. *abscessus* subsp. *massiliense* strains is higher than that among *M*. *abscessus* subsp. *abscessus* strains.

In conclusion, we identified two *M*. *abscessus* subsp. *massiliense* rough strains from Korean patients with *hsp65* genes that might have been laterally transferred from *M*. *abscessus* subsp. *abscessus*. To the best of our knowledge, this is the first report to demonstrate LGT events associated with the *hsp65* gene in mycobacteria. This report also suggests that there is potential for misidentification when *hsp65*-based protocols are used for mycobacterial identification.

## Supporting information

S1 Table Primer sets used for PCR amplification and sequencing in this study. (DOCX)

S2 Table GenBank accession numbers corresponding to the sequences obtained in this study. (DOCX)

S3 Table Sequence similarities of the *hsp65*, *rpoB*, and 7 MLST genes and concatenated sequences among *M*. *abscessus* strains. (DOCX)

S1 Fig Alignment of the complete *hsp65* gene sequences of *M*. *abscessus* strains and the Asan 55262 strain. (DOCX)
