# Supplementary material for: New Mycobacteroides abscessus subsp. massiliense strains with recombinant hsp65 gene laterally transferred from Mycobacteroides abscessus subsp. abscessus: Potential for misidentification of M. abscessus strains with the hsp65-based method
Source: PLoS One. 2019 Sep 13;14(9):e0220312. doi: 10.1371/journal.pone.0220312 (PMC6743754; doi:10.1371/journal.pone.0220312)
Supplement: S1 Table — (DOCX) [file pone.0220312.s001.docx]

**S1 Table.** Primer sets used for PCR amplification and sequencing in this study

| Genes | Primer | Primer sequence (5' to 3') | Amplicon size (bp) |
| --- | --- | --- | --- |
| *argH* | F | GACGAGGGCGACTTC | 629 |
|  | R | GTGCGCGAGCAGATGATG |  |
| *cya* | F | GTGAAGCGGGCCAAGAAG | 647 |
|  | R | AACTGGGAGCCCAGGAGC |  |
| *erm*(41) | F | GACCGGGGCCTTCTTCGTGATC | 397 or 673 |
|  | R | AGCTTCCCCGCACCGATTCCA |  |
| *glpK* | F | AATCTCACCGGCGGTGTC | 609 |
|  | R | GGACAGACCCACGATGGC |  |
| *gnd* | F | GTGACGTCGGAGTGGG | 634 |
|  | R | CTTCGCCTCAGGTCAGCTC |  |
| *hsp65* (partial) | F | ATCGCCAAGGAGATCGAGCT | 644 |
|  | R | AAGGTGCCGCGGATCTTCTT |  |
| *murC* | F | CGGACGAAAGCGACGGCT | 607 |
|  | R | CCAAAACCCTGCTGAGCC |  |
| *purH* | F | CGGAGGCTTCACCCTGGA | 634 |
|  | R | CAGGCCACCGCTGATCTG |  |
| *pta* | F | GATCGGGCGTCATGCCCT | 720 |
|  | R | ACGAGGCACTGCTCTCCC |  |
| *rpoB* | F | ATCGCCGACGGTCCCTGC | 1092 |
|  | R | GAACCGCTGGCCACCGAACT |  |
| *hsp65* (full) | 1F | AAGGCTGCGCGATAGATACG | 748 |
|  | 1R | CGATGCGGACGCCG |  |
|  | 2F | GACAAGCGCGACACCGT | 684 |
|  | 2R | CAGCGCCGACAGACGAC |  |
|  | 3F | CGTCGTCTGTCGGCGC | 684 |
|  | 3R | CGTCCACGATGGGCTTCT |  |
|  | 4F | CCAGAAGCCCATCGTGGA | 933 |
|  | 4R | CCGCGCCTTGCCG |  |
|  | 5F | AACCGGGACGGCGAG | 943 |
|  | 5R | TGCAGATCCTTCGGTGCC |  |
