# Supplementary material for: New Mycobacteroides abscessus subsp. massiliense strains with recombinant hsp65 gene laterally transferred from Mycobacteroides abscessus subsp. abscessus: Potential for misidentification of M. abscessus strains with the hsp65-based method
Source: PLoS One. 2019 Sep 13;14(9):e0220312. doi: 10.1371/journal.pone.0220312 (PMC6743754; doi:10.1371/journal.pone.0220312)
Supplement: S2 Table — (DOCX) [file pone.0220312.s002.docx]

**S2 Table.** GenBank accession numbers corresponding to obtained sequences in this study

|  | Strains | |
| --- | --- | --- |
| Genes | Asan 55184 | Asan 55262 |
| *argH* | MH430895 | MH430896 |
| *cya* | MH430897 | MH430898 |
| *glpK* | MH430899 | MH430900 |
| *gnd* | MH430901 | MH430902 |
| *murC* | MH430903 | MH430904 |
| *pta* | MH430905 | MH430906 |
| *purH* | MH430907 | MH430908 |
| *hsp65*(603 bp) | MH430909 | MH430910 |
| *rpoB* (711 bp) | MH430911 | MH430912 |
| Total *hsp65* | - | MH430913 |
