# Supplementary material for: New Mycobacteroides abscessus subsp. massiliense strains with recombinant hsp65 gene laterally transferred from Mycobacteroides abscessus subsp. abscessus: Potential for misidentification of M. abscessus strains with the hsp65-based method
Source: PLoS One. 2019 Sep 13;14(9):e0220312. doi: 10.1371/journal.pone.0220312 (PMC6743754; doi:10.1371/journal.pone.0220312)
Supplement: S3 Table — (DOCX) [file pone.0220312.s003.docx]

**S3 Table.** Sequence similarities of *hsp65*, *rpoB*, 7 MLST genes and concatenated sequences among *M. abscessus* strains

*hsp65* (603 bp)

| Strains | Sequence similarities (%) | | | | | |
| --- | --- | --- | --- | --- | --- | --- |
|  | Mabs | Mmass | Mbol | 50594 | 55184 | 55262 |
| Mabs |  | 98.8 | 98.8 | 98.5 | 99.8 | 99.8 |
| Mmass |  |  | 99.3 | 99.7 | 98.7 | 98.7 |
| Mbol |  |  |  | 99 | 98.7 | 98.7 |
| 50594 |  |  |  |  | 98.3 | 98.3 |
| 55184 |  |  |  |  |  | 100 |
| 55262 |  |  |  |  |  |  |

*argH* (503 bp)

| Strains | Sequence similarities (%) | | | | | |
| --- | --- | --- | --- | --- | --- | --- |
|  | Mabs | Mmass | Mbol | 50594 | 55184 | 55262 |
| Mabs |  | 95.4 | 96.6 | 96.2 | 95.4 | 95.4 |
| Mmass |  |  | 97 | 99 | 100 | 100 |
| Mbol |  |  |  | 98.2 | 97 | 97 |
| 50594 |  |  |  |  | 99 | 99 |
| 55184 |  |  |  |  |  | 100 |
| 55262 |  |  |  |  |  |  |

*cya* (541 bp)

| Strains | Sequence similarities (%) | | | | | |
| --- | --- | --- | --- | --- | --- | --- |
|  | Mabs | Mmass | Mbol | 50594 | 55184 | 55262 |
| Mabs |  | 98.2 | 98.2 | 98.2 | 98.2 | 98.2 |
| Mmass |  |  | 97.4 | 100 | 100 | 100 |
| Mbol |  |  |  | 97.4 | 97.4 | 97.4 |
| 50594 |  |  |  |  | 100 | 100 |
| 55184 |  |  |  |  |  | 100 |
| 55262 |  |  |  |  |  |  |

| *glpK* (563 bp) | |  |  |  |  |  |
| --- | --- | --- | --- | --- | --- | --- |
| Strains | Sequence similarities (%) | | | | | |
|  | Mabs | Mmass | Mbol | 50594 | 55184 | 55262 |
| Mabs |  | 98 | 98.6 | 98 | 97.7 | 97.9 |
| Mmass |  |  | 98.9 | 100 | 99.6 | 99.8 |
| Mbol |  |  |  | 98.9 | 98.6 | 98.8 |
| 50594 |  |  |  |  | 99.6 | 99.8 |
| 55184 |  |  |  |  |  | 99.8 |
| 55262 |  |  |  |  |  |  |

| *gnd* (494 bp) | |  |  |  |  |  |
| --- | --- | --- | --- | --- | --- | --- |
| Strains | Sequence similarities (%) | | | | | |
|  | Mabs | Mmass | Mbol | 50594 | 55184 | 55262 |
| Mabs |  | 97.6 | 95.5 | 97.8 | 97.8 | 97.8 |
| Mmass |  |  | 96.8 | 99.8 | 99.8 | 99.8 |
| Mbol |  |  |  | 97 | 97 | 97 |
| 50594 |  |  |  |  | 100 | 100 |
| 55184 |  |  |  |  |  | 100 |
| 55262 |  |  |  |  |  |  |

| *murC* (545 bp) | |  |  |  |  |  |
| --- | --- | --- | --- | --- | --- | --- |
| Strains | Sequence similarities (%) | | | | | |
|  | Mabs | Mmass | Mbol | 50594 | 55184 | 55262 |
| Mabs |  | 98 | 96.5 | 98 | 98 | 98 |
| Mmass |  |  | 96.7 | 100 | 99.6 | 99.6 |
| Mbol |  |  |  | 96.7 | 97.1 | 97.1 |
| 50594 |  |  |  |  | 99.6 | 99.6 |
| 55184 |  |  |  |  |  | 100 |
| 55262 |  |  |  |  |  |  |

| *pta* (486 bp) | |  |  |  |  |  |
| --- | --- | --- | --- | --- | --- | --- |
| Strains | Sequence similarities (%) | | | | | |
|  | Mabs | Mmass | Mbol | 50594 | 55184 | 55262 |
| Mabs |  | 98.1 | 98.4 | 98.1 | 97.9 | 97.9 |
| Mmass |  |  | 97.7 | 100 | 99.8 | 99.8 |
| Mbol |  |  |  | 97.7 | 97.5 | 97.5 |
| 50594 |  |  |  |  | 99.8 | 99.8 |
| 55184 |  |  |  |  |  | 100 |
| 55262 |  |  |  |  |  |  |

| *purH* (549 bp) | |  |  |  |  |  |
| --- | --- | --- | --- | --- | --- | --- |
| Strains | Sequence similarities (%) | | | | | |
|  | Mabs | Mmass | Mbol | 50594 | 55184 | 55262 |
| Mabs |  | 98.2 | 98 | 97.4 | 97.6 | 97.6 |
| Mmass |  |  | 99.8 | 97.4 | 97.6 | 97.6 |
| Mbol |  |  |  | 97.3 | 97.4 | 97.4 |
| 50594 |  |  |  |  | 99.1 | 99.1 |
| 55184 |  |  |  |  |  | 100 |
| 55262 |  |  |  |  |  |  |

| Concatenation (7 MLST genes) (3,681 bp) | | | |  |  |  |
| --- | --- | --- | --- | --- | --- | --- |
| Strains | Sequence similarities (%) | | | | | |
|  | Mabs | Mmass | Mbol | 50594 | 55184 | 55262 |
| Mabs |  | 97.7 | 97.4 | 97.7 | 97.6 | 97.6 |
| Mmass |  |  | 97.9 | 99.5 | 99.5 | 99.5 |
| Mbol |  |  |  | 97.6 | 97.5 | 97.5 |
| 50594 |  |  |  |  | 99.6 | 99.6 |
| 55184 |  |  |  |  |  | 100 |
| 55262 |  |  |  |  |  |  |

| Concatenation (7 MLST + *hsp65* genes) (4,284 bp) | | | |  |  |  |
| --- | --- | --- | --- | --- | --- | --- |
| Strains | Sequence similarities (%) | | | | | |
|  | Mabs | Mmass | Mbol | 50594 | 55184 | 55262 |
| Mabs |  | 97.9 | 97.6 | 97.8 | 97.9 | 97.9 |
| Mmass |  |  | 98.1 | 99.5 | 99.4 | 99.4 |
| Mbol |  |  |  | 97.8 | 97.7 | 97.7 |
| 50594 |  |  |  |  | 99.4 | 99.4 |
| 55184 |  |  |  |  |  | 100 |
| 55262 |  |  |  |  |  |  |

| Concatenation (7 MLST + *hsp65* + *rpoB* genes) (4,995 bp) | | | | |  |  |
| --- | --- | --- | --- | --- | --- | --- |
| Strains | Sequence similarities (%) | | | | | |
|  | Mabs | Mmass | Mbol | 50594 | 55184 | 55262 |
| Mabs |  | 97.7 | 97.4 | 97.6 | 97.7 | 97.7 |
| Mmass |  |  | 98.1 | 99.5 | 99.4 | 99.4 |
| Mbol |  |  |  | 97.9 | 97.8 | 97.8 |
| 50594 |  |  |  |  | 99.5 | 99.5 |
| 55184 |  |  |  |  |  | 100 |
| 55262 |  |  |  |  |  |  |

Mabs, *M. abscessus* subsp. *abscessus* ATCC 19977^T^; Mmass, *M. abscessus* subsp. *massiliense* CCUG 48898; Mbol, *M. abscessus* subsp. *bolletii* CIP 108541^T^.
