# Supplementary figures and images for: New Mycobacteroides abscessus subsp. massiliense strains with recombinant hsp65 gene laterally transferred from Mycobacteroides abscessus subsp. abscessus: Potential for misidentification of M. abscessus strains with the hsp65-based method
Source: PLoS One. 2019 Sep 13;14(9):e0220312. doi: 10.1371/journal.pone.0220312 (PMC6743754; doi:10.1371/journal.pone.0220312)

**Fig S1.** Alignment of the complete *hsp65* gene sequences of *M. abscessus* strains and Asan 55262 strain.


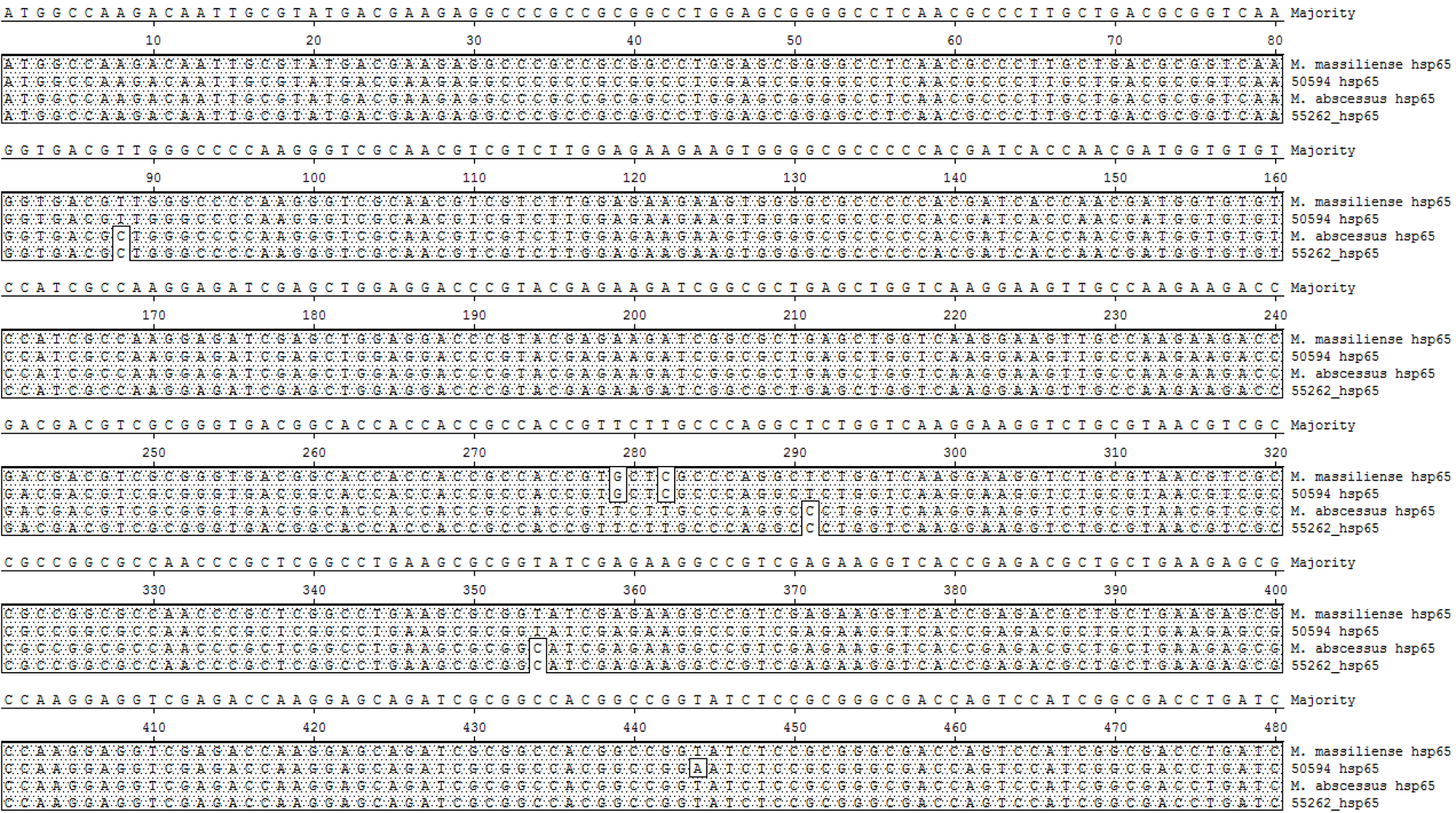


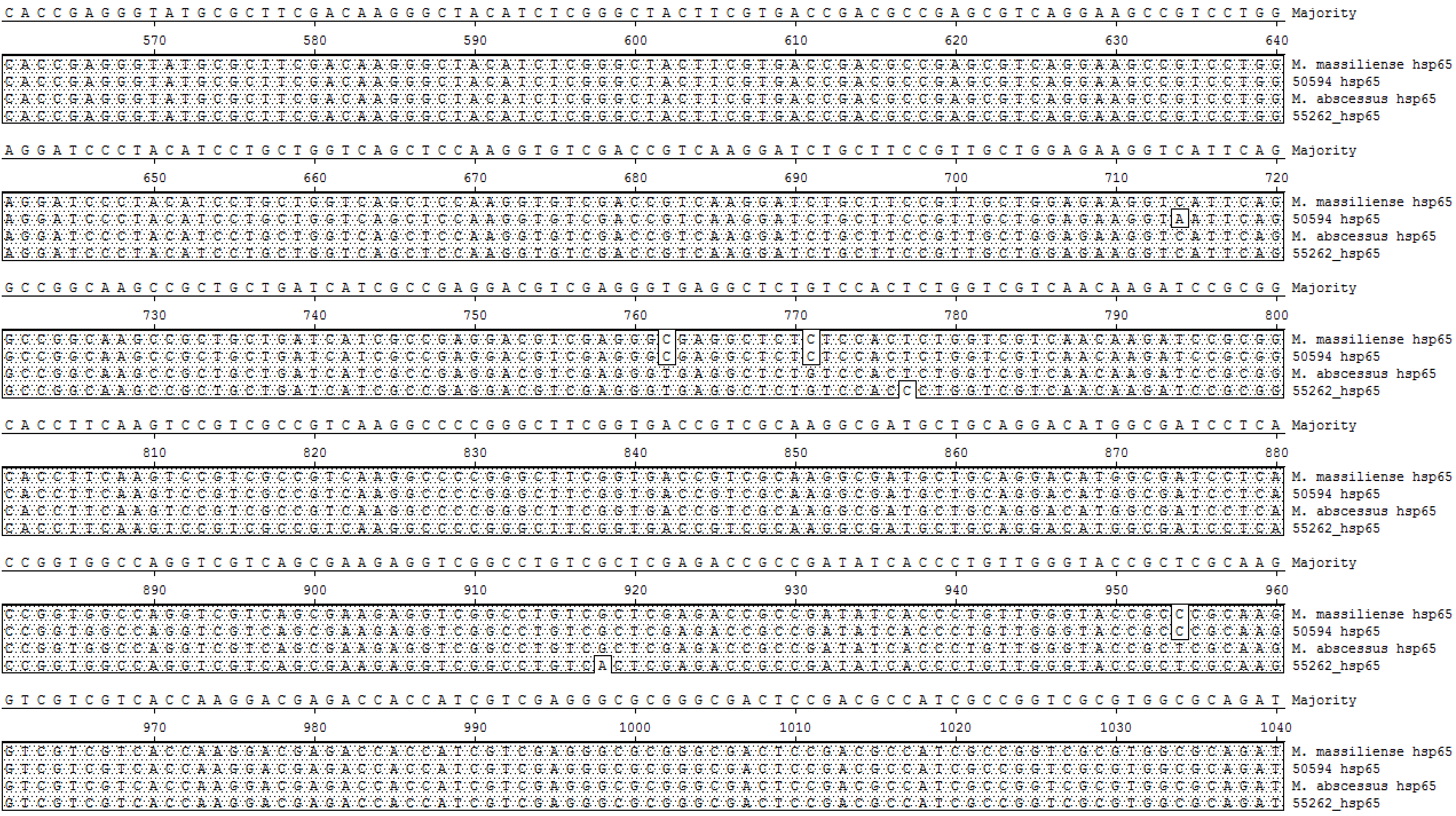


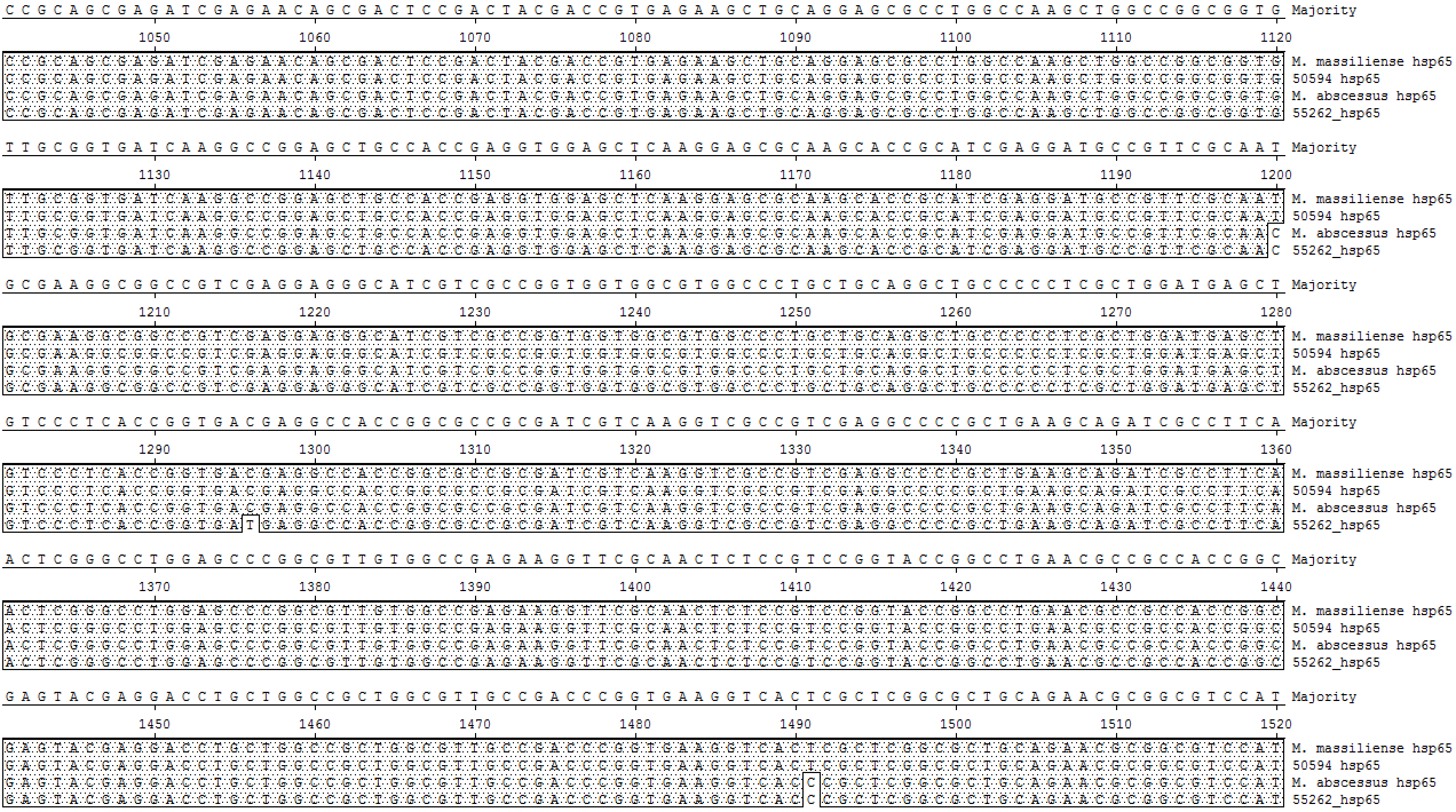


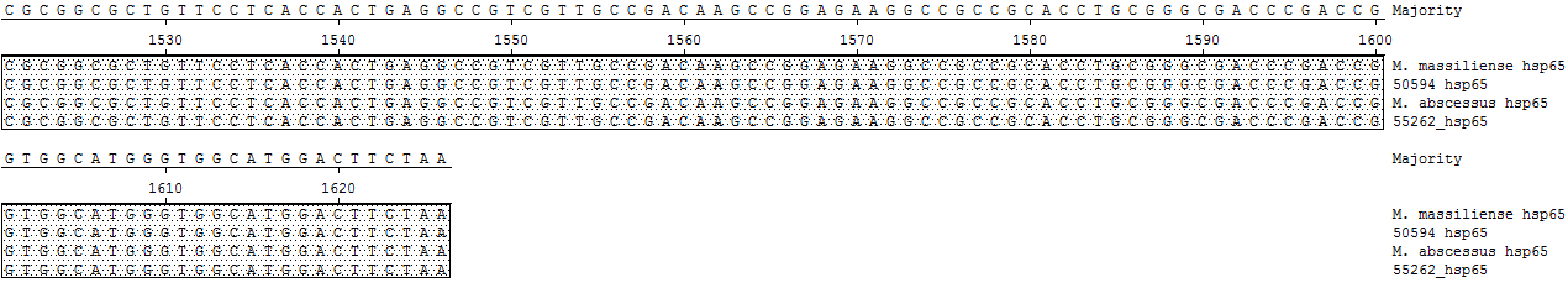

Supplement: S1 Fig — (DOCX) [file pone.0220312.s004.docx]
